# Pleural thickening on screening chest X-rays: a single institutional study

**DOI:** 10.1186/s12931-019-1116-9

**Published:** 2019-07-05

**Authors:** Akira Saito, Yukichika Hakamata, Yukiko Yamada, Mitsuhiro Sunohara, Megumi Tarui, Yoko Murano, Akihisa Mitani, Kimie Tanaka, Takahide Nagase, Shintaro Yanagimoto

**Affiliations:** 1 Department of Respiratory Medicine, Graduate School of Medicine, The University of Tokyo, 7-3-1 Hongo, Bunkyo-ku, Tokyo, 113-0033 Japan; 2 Division for Health Service Promotion, The University of Tokyo, 7-3-1 Hongo, Bunkyo-ku, Tokyo, 113-0033 Japan

**Keywords:** Chest X-ray, Screening, Pleural thickening, Pulmonary apical cap, Body mass index

## Abstract

Although pleural thickening is a common finding on routine chest X-rays, its radiological and clinical features remain poorly characterized. Our investigation of 28,727 chest X-rays obtained from annual health examinations confirmed that pleural thickening was the most common abnormal radiological finding. In most cases (92.2%), pleural thickening involved the apex of the lung, particularly on the right side; thus, it was defined as a pulmonary apical cap. Pleural thickening was more common in males than in females and in current smokers or ex-smokers than in never smokers. The prevalence increased with age, ranging from 1.8% in teenagers to 9.8% in adults aged 60 years and older. Moreover, pleural thickening was clearly associated with greater height and lower body weight and body mass index, suggesting that a tall, thin body shape may predispose to pleural thickening. These observations allowed us to speculate about the causative mechanisms of pleural thickening that are attributable to disproportionate perfusion, ventilation, or mechanical forces in the lungs.

**Electronic supplementary material:**

The online version of this article (10.1186/s12931-019-1116-9) contains supplementary material, which is available to authorized users.

## Background

Pleural thickening is a common finding on routine chest X-rays. It typically involves the apex of the lung, which is called ‘pulmonary apical cap’. On chest X-rays, the apical cap is an irregular density located at the extreme apex and is less than 5 mm in width [1]. In the early twentieth century, a pulmonary apical cap was thought to be a tubercular lesion; however, detailed pathological studies conducted in the 1970s found no evidence of tuberculosis [2–4]. The apical cap is a fibroelastic scar involving the visceral pleura and lung parenchyma at the apex and is occasionally observed in healthy and asymptomatic individuals [5].

In 1974, Renner et al. [3] identified unilateral or bilateral apical cap shadows in 22.1% (*n* = 57) of 258 routine chest X-rays. This was a pioneering radiological study; however, the sample size was small. Surprisingly, no subsequent studies have investigated the prevalence of an apical cap on chest X-ray examination [3, 6] or its association with various subject characteristics.

Pleural thickening may be a manifestation of several pulmonary diseases, including mycobacterial infection, lung cancer, and idiopathic interstitial pneumonia. Of particular note, pleuroparenchymal fibroelastosis is increasingly recognized as a rare form of idiopathic interstitial pneumonia characterized by pleural and subjacent parenchymal fibrosis predominantly in the upper lobes, which may mimic an apical cap. Therefore, clinicians must rule out these pathological conditions when evaluating pleural thickening found on a chest X-ray. Moreover, it is necessary to characterize the radiological and clinical features of pleural thickening or an apical cap that is not disease-related in and of itself.

We reviewed 28,727 chest X-rays obtained from annual health examinations performed between April 2017 and March 2018 in a large population. We confirmed that pleural thickening, typically located at the apex of the lung, was the most common abnormal finding. Furthermore, we investigated the prevalence and laterality of pleural thickening and its association with subject characteristics, including sex, age, smoking status, height, body weight, and body mass index (BMI). Our findings suggest that individuals with taller and thinner body shapes may be prone to pleural thickening. Given these findings, we speculate that an apical cap may be the result of disproportionate perfusion, ventilation, or mechanical forces in the lungs.

## Methods

### Study population

We conducted a cross-sectional analysis of chest X-rays obtained from the annual health examinations of 28,727 employees and students at the University of Tokyo between April 2017 and March 2018. The chest X-rays were assessed independently by two physicians. Abnormal findings were referred to board-certified pulmonologists (M.S., M.T., Y.M., A.M., and A.S.) for further evaluation using available clinical information and previous X-ray images.

### Pleural thickening assessment

Four areas of the lung (apical, upper, middle, and lower portions) were examined bilaterally for the presence of pleural thickening. Pleural thickening that involved the apex of either lung was defined as an apical cap.

### Statistical analyses

Between-group comparisons were made using two-tailed Student’s *t*-tests, and the chi-square test was used to assess the associations of pleural thickening with sex and smoking status. The binary logistic regression model was used to evaluate the effects of sex, age, smoking status, and BMI on pleural thickening. All statistical tests were performed using the Statistical Package for the Social Sciences (SPSS Inc., Chicago, IL, USA).

## Results

We investigated the prevalence and distribution of pleural thickening and its association with subject characteristics, including sex, age, smoking status, height, body weight, and BMI.

### Pleural thickening is the most common abnormal finding on screening chest X-rays

**Fig. 1:**
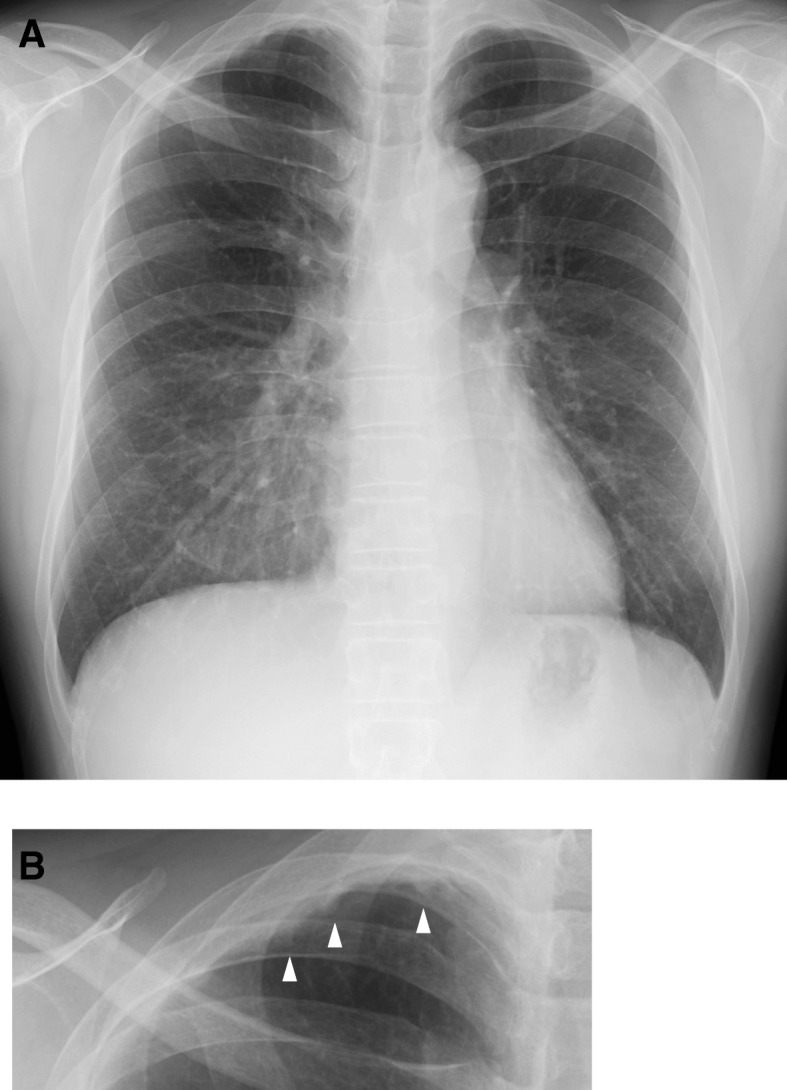
Representative chest X-ray image of pleural thickening. **a**. Chest X-ray image of a healthy 47-year-old male. The right apical cap appears as an irregular, wedge-shaped density. **b**. Enlarged image of the right apex. The white triangles indicate pleural thickening

Chest X-rays obtained from the annual health examinations of 28,727 individuals between April 2017 and March 2018 were independently reviewed by two physicians. The study included 10,012 females and 18,715 males, and the mean (± standard deviation) age was 30.5 ± 12.1 (range, 17–83) years. Four areas of the lung (apical, upper, middle, and lower portions) were examined bilaterally for the presence of abnormal findings. There could be more than one finding in a subset of individuals. In total, 4041 abnormal findings were observed on the chest X-rays of 3113 individuals by at least one physician (Additional file 1: Table S1). Pleural thickening was the most common finding (35.2%; *n* = 1423) that was identified in 911 individuals. A representative X-ray image of pleural thickening is shown in Fig. 1. The prevalence of pleural thickening was 3.2% (*n* = 911/28,727) in our sample.

### Pleural thickening was found predominantly at the apex of the right lung

**Fig. 2:**
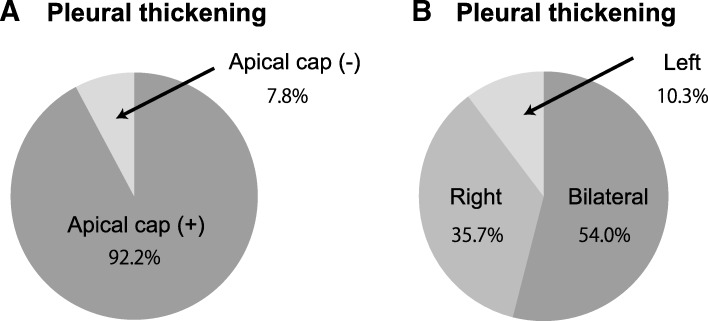
Pleural thickening distribution. **a**. Pie chart showing the percentages of pleural thickening cases with and without an apical cap. **b**. Pie chart showing the percentages of bilateral and unilateral pleural thickening cases

The apex of the lung was the most frequently affected area (Additional file 1: Table S2). Pleural thickening involving the apical area of either lung was defined as an apical cap, which accounted for 92.2% (*n* = 836/907) of the cases (Fig. 2a). More than half of the cases were bilateral and 35.7% involved thickening on the right side only. Together, the bilateral and right-sided cases comprised nearly 90% of the 907 cases (Fig. 2b). These findings indicate that the pleural thickening and apical cap cases overlapped, and that pleural thickening occurred predominantly in the right lung (Fig. 2).

### Pleural thickening was more common in males and increased with age

**Fig. 3:**
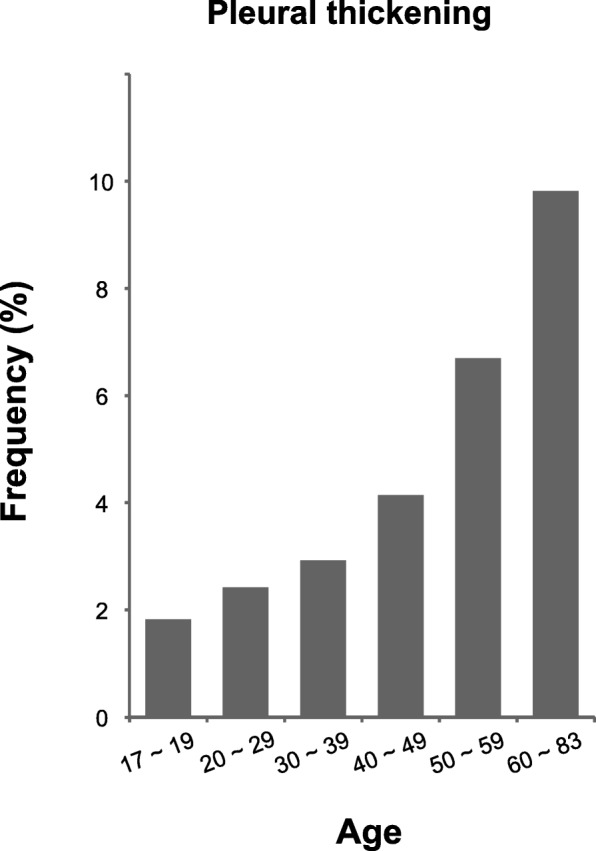
Frequencies of pleural thickening according to age

Pleural thickening was more common in males (3.4%) than in females (2.7%; *p* < 0.01) (Additional file 1: Table S3), and the incidence increased with age, ranging from 1.8% in teenagers (17–19 years) to 9.8% in adults aged 60–83 years (Additional file 1: Table S4). It is worth noting that pleural thickening occurred in individuals as young as 18 years, and the prevalence increased markedly after the age of 40 years (Fig. 3).

### Pleural thickening was more common in current and ex-smokers than in never smokers

We further investigated whether smoking history was associated with pleural thickening. Information about smoking status was available for 25,291 individuals (16,043 males and 9248 females). The smoking rates of those with and without pleural thickening were 6.3 and 4.7%, respectively. The percentages of individuals with a smoking history (i.e., current smokers and ex-smokers) with and without pleural thickening were 14.6 and 10.2%, respectively. The prevalence of pleural thickening was higher in current smokers (4.4%) and ex-smokers (5.0%) than in never smokers (3.2%; *p* < 0.01) (Additional file 1: Table S5). Moreover, these trends were confirmed when males and females were analyzed separately.

### Pleural thickening was associated with greater height and lower body weight and BMI

**Fig. 4:**
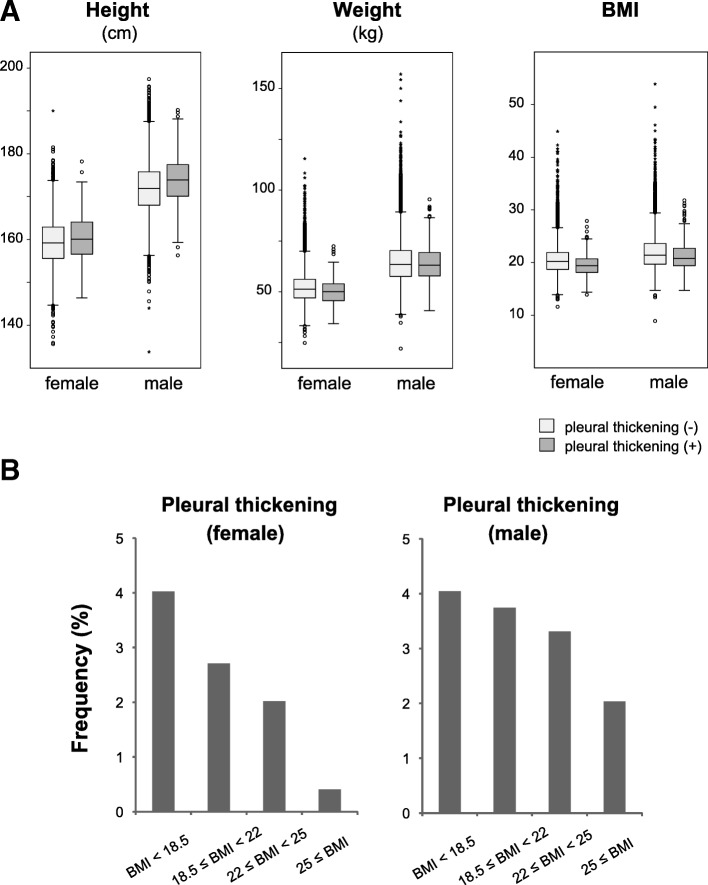
Associations of pleural thickening with height, body weight, and BMI. **a**. Box plot showing the height (cm), body weight (kg), and BMI in individuals with and without pleural thickening. The line in the middle of the box indicates the median with the top and bottom ends of the box indicating the 75th and 25th percentiles, respectively. The circles and asterisks indicate outliers and extreme outliers, respectively. **b**. Frequencies of pleural thickening according to BMI values

Next, we investigated the association between pleural thickening and body shape. Height, body weight, and BMI were compared in individuals with and without pleural thickening (Fig. 4a). Males and females with pleural thickening were taller, weighed less, and had lower BMI than those without pleural thickening (*p* < 0.01 for each comparison). Further comparisons of sex, BMI, and pleural thickening revealed that underweight individuals (BMI < 18.5) had the highest percentage of pleural thickening, which was approximately 4% in both males and females (Additional file 1: Table S6). Conversely, the frequency of pleural thickening was lowest among overweight or obese individuals (25 ≤ BMI). The tendency toward an association between higher BMI and lower frequency of pleural thickening was more prominent in females (Fig. 4b). These findings indicate that greater height and lower body weight and BMI predispose to pleural thickening, suggesting that the causative mechanism is related to a tall, thin body shape.

Finally, a binary logistic regression analysis was performed to evaluate the effects of sex, age, smoking status, and BMI on the likelihood of pleural thickening. We analyzed 25,286 cases with available information of all these characteristics, and odds ratios (OR) with 95% confidence intervals (CI) were calculated. As a result, male sex (OR = 2.042, 95% CI 1.738–2.399), age (OR = 1.051, 95% CI 1.045–1.057), and BMI (OR = 0.836, 95% CI 0.814–0.860) were found to be related to pleural thickening with statistical significance.

## Discussion

Although a pulmonary apical cap has been recognized as a non-specific fibrotic change at the apex of the lung for half a century [6], only a few studies with small sample sizes have investigated the prevalence of an apical cap in the general population and the associations with clinical features [2–4]. Our study of 28,727 individuals confirmed that pleural thickening was the most common finding on routine chest X-rays (Additional file 1: Table S1). We found that more than 90% of the cases involved apical pleural thickening, or an apical cap (Fig. 2). The prevalence of pleural thickening increased with age: the percentages of cases in individuals in their 20s, 30s, 40s, and 50s were 2.4, 2.9, 4.1, and 6.7%, respectively (Fig. 3). It is worth noting that pleural thickening (an apical cap, in most cases) was found in 1.8% of subjects under the age of 20 years, suggesting that pleural thickening is not simply an aging phenomenon. Healthcare providers should consider these findings when reviewing routine chest X-rays.

A limitation of our study is that the sample was drawn from a single academic institution, the University of Tokyo, and consisted primarily of Japanese students and university employees (> 90%). Additional studies are needed to examine ethnic and socioeconomic differences in the prevalence of pleural thickening and an apical cap. Of the 16,043 males in our study, 6.2% (*n* = 1001) were current smokers and 6.7% (*n* = 1072) were ex-smokers, whereas 2.2% (*n* = 204) of the 9248 females were current smokers and 3.7% (*n* = 339) were ex-smokers. These figures are considerably lower than the nationwide smoking rates in Japan in 2017 (29.4% in males and 7.2% in females) or those reported in the United States in 2016 (17.5% in males and 13.5% in females) [7, 8]. Given our finding that the frequency of an apical cap was higher in individuals with a smoking history compared with never smokers (Additional file 1: Table S5), pleural thickening may be more prevalent in the general population with higher smoking rates than in our study population.

Another limitation of our study population is the lack of detailed information about past medical history and medication. Further studies are necessary to evaluate the possible association of pleural thickening with comorbid diseases.

To date, few studies have investigated the pathological features of an apical cap [2–5]. It is generally accepted that an apical cap is a distinct fibroelastic plaque in the lung that contains mature collagen and elastin fibers [6]. In 1970, Butler et al. [2] examined 48 autopsy lung specimens and noted mural thickening in the small muscular arteries subjacent to the apical cap and chronic bronchitis in more than half of the cases. Based on these observations, the authors concluded that an apical cap is a localized parenchymal lesion, which is presumably the result of persistent or repeated inflammation. They further postulated that the relatively decreased perfusion at the apex of the lung may impede the resolution of inflammation. More recently, Yousem [5] reviewed 13 surgically resected lung specimens with an apical cap and reported consistent pathological findings suggesting chronic ischemia as a major cause of an apical cap [5].

Due to the effects of gravity, ventilation and perfusion rates are lowest at the apex and highest at the base of the lung in the upright position [9]. Furthermore, the ventilation/perfusion ratio is highest at the apex because ventilation is relatively greater than perfusion compared with other lung areas [9]. As such, it seems reasonable that the apex is more susceptible to chronic ischemia, which may explain why pleural thickening is found predominantly at the apex and in the upper portion of the lung (Fig. 2). Moreover, lower ventilation and perfusion at the apex may increase the risk of sustained exposure to pathogens or environmental irritants that can trigger the inflammation associated with pleural thickening. Furthermore, intrapleural pressure is more negative, and transpulmonary pressure is greater at the apex than in the lower portion of the lung [9]. Therefore, mechanical forces generated by repeated cycles of respiration may be greater at the apex, which may in turn promote the fibrotic response [10]. However, these pathogenic mechanisms remain a matter of speculation.

Importantly, we found that pleural thickening (primarily an apical cap) was associated with greater height and lower body weight and BMI (Fig. 4). We suggest two possible explanations for this finding. Given that the lungs are proportional to body mass, the anatomical and pathophysiological differences between the apex and lower portion of the lung may be greater among taller, thinner individuals leading to an increased risk of inflammation and/or ischemia at the apex. Alternatively, individuals with a genetic predisposition for greater height or lower BMI may be more prone to developing pleural thickening [11]. Future genome-wide association studies of pleural thickening or apical cap may clarify this issue.

We found that pleural thickening occurred predominantly in the right lung. Anatomical differences may account for this finding. The left lung has two lobes and thus a lower volume than the right lung, which has three lobes, and the heart is located on the left side. Conceivably, there may be a greater ventilation/perfusion mismatch and stronger mechanical forces acting at the apex of the right lung than at the left lung.

Pleuroparenchymal fibroelastosis is a rare form of idiopathic interstitial pneumonia that affects the visceral pleura and subpleural parenchyma with upper lobe predominance. Although no case of pleuroparenchymal fibroelastosis was identified in our study, it is conceivable that an early lesion of pleuroparenchymal fibroelastosis mimics an apical cap on chest X-rays. Intriguingly, a previous study found that the BMI in patients with pleuroparenchymal fibroelastosis was significantly lower than that of patients with idiopathic pulmonary fibrosis, a common form of idiopathic interstitial pneumonia with lower lobe predominance (mean BMI 18.6 vs. 25.1, respectively) [12]. Moreover, another study found that the histological findings for pleuroparenchymal fibroelastosis were “strikingly similar” to those for pulmonary apical cap [6]. Thus, it is tempting to speculate that a similar causative mechanism related to tall, thin body shapes may be involved in both apical cap and pleuroparenchymal fibroelastosis.

## Conclusion

Pleural thickening was the most common finding on routine chest X-ray examinations and more than 90% of the cases were defined as a pulmonary apical cap with right lung predominance. Pleural thickening increased with age and was more frequent in males and smokers. We found that pleural thickening was associated with greater height and lower body weight and BMI. It may be that tall, thin body shapes exaggerate disproportionate perfusion, ventilation, or mechanical forces in the lungs, which may increase the risk of ischemia and/or a fibrotic response at the apex of the lung.

## Additional file

Additional file 1: **Table S1.** Top 10 most common abnormal findings on screening chest X-rays. **Table S2.** Distribution and laterality of pleural thickening. **Table S3.** Pleural thickening and sex. **Table S4.** Pleural thickening and age distribution. **Table S5.** Pleural thickening and smoking status. **Table S6.** Pleural thickening and BMI. (XLSX 18 kb)

## Data Availability

Not applicable.

## Abbreviation

BMI: body mass index
